# I feel you-monitoring environmental variables related to asthma in an integrated real-time frame

**DOI:** 10.1186/s13104-015-1421-4

**Published:** 2015-09-11

**Authors:** Anabela Gonçalves Berenguer

**Affiliations:** Human Genetics Laboratory, University of Madeira, 9000-390 Funchal, Portugal; Department of Computer Science and Engineering, University of Oulu, PL 4500, 90014 Oulu, Finland

**Keywords:** Asthma, Genetics, Environment, Epigenetics, Real-time monitoring, Sensors, Omics, Exposome

## Abstract

The study of asthma and other complex diseases has proven to be a “moving target” for researchers due to its complex aetiology, difficulty in definition, and immeasurable environmental effects. A large number of studies regarding the contribution of both genetic and environmental factors often result in contradictory results, in part due to the highly heterogeneous nature of asthma. Recent literature has focused on the epigenetic signatures of asthma caused by environmental factors, highlighting the importance of environment. However, unlike the genetic techniques, environmental assessment still lacks accuracy. A plausible solution for this problem would be an individual-based environmental exposure assessment, relying on new technologies such as personal real-time environmental sensors. This could prove to enable the assessment of the whole environmental exposure—or exposome—matching in terms of precision the genome that is emphasized in most studies so far. In addition, the measurement of the whole array of biological molecules, in response to the environment action, could help understand the context of the disease. The current perspective comprises a beyond-genetics integrated vision of omics technology coupled with real-time environmental measures targeting to enhance our comprehension of the disease genesis.

## Discussion

Asthma is a complex disease influenced by the interaction of multiple environmental and genetic factors. It is defined as a chronic inflammation that narrows the airways, causing airflow obstruction, leading to wheezing, chest tightness and coughing [1]. The disease affects more than 235 million people worldwide [2] and it was responsible for 255,000 deaths in 2005, according to the World Health Organization [3].

Asthma is an ever-growing pathology especially in the westernized world, and a large number of studies assessing the contribution of both genetic and environmental factors to the disease aetiology have been reported [4–8].

However, the results are often contradictory. As an example, the association of the polymorphism *C*-*589T* in the promoter region of the interleukin 4 gene (*IL4*) (rs2243250) to asthma has been inconsistent across different populations, despite its biological plausibility as a candidate gene [9]. On the other hand, the *GSDML-236* (rs7216389) SNP, whose biological role was unknown at the time of its discovery, has been successfully replicated as associated to different asthma phenotypes across a number of studies [10–20].

But even among statistically robust GWA studies, non-replication and inconsistencies are still an issue [21]. In fact, GWA analyses may not capture all relevant variation, such as rare variants thought to have larger phenotypic effects than common variants. In addition, GWAs statistical approach may not be adequate to model the polygenic nature of asthma as well as its gene –environment interactions [22]. Similarly, on the environmental aspect, the effect of motor air pollution on asthma in children has also produced conflicting results [23]. Distinct study designs, dissimilar asthma definitions, different environmental setting and diverse genetic backgrounds are amongst the main explanations for the lack of consistency between studies [24–26].

Moreover, asthma is a highly heterogeneous disease where various phenotypes can be defined, such as early-onset allergic asthma, late-onset eosinophilic, exercise-induced, obesity related and neutrophilic asthma [27]. Asthma endotypes have also been proposed as a way to break down the disease according to its pathophysiological mechanism. For instance, within a specific phenotype, as obesity-related asthma, a number of endotypes can emerge (airflow obstruction caused by obesity, severe steroid-dependent asthma, severe late-onset hypereosinophilic asthma, etc.) [28]. But, again, within an endotype, the disease severity can vary [28] adding to the complexity of the definition. Under these circumstances, new approaches for understanding the disease are needed.

We have recently proposed that rather than as a snapshot, asthma should be considered as a motion picture across evolution and in permanent interaction with its surrounding environment [29].

This becomes particularly relevant when one considers two key aspects: the significant increase of asthma in developed societies over the last two decades, and the epigenetic effects of certain environmental exposure over asthma.

Numerous examples of epigenetic effects over DNA, as a result of air pollution, have recently appeared in literature. For example, methylation levels in the promoter of *NPSR1* gene, relate to asthma in children and adults as a result of smoking exposure [30]. High methylation levels in the 5′ UTR of *ADRB2* gene also relate to severe childhood asthma, in association with indoor exposure to NO_2_ [31]. Particulate matter with aerodynamic diameter ≤2.5 μm (PM2.5) has been found to influence iNOS methylation pattern, affecting FeNO levels, a predictor for the future risk of asthma and wheeze [32]. Methylation patterns in a number of genes included in the asthma pathway were found associated to specific airborne particulate matter like black carbon (*HLA*-*DOB*, *FCER1A*, *IL9*, and *PRG2*), sulphate (*HLA*-*DPA1*, *IL*-*10*, *RNASE3*, *CCL11*) or both (*FCER1G*) [33]. Exposure to inhaled diesel particles and allergen have been found to induce hypomethylation within a CpG^−408^ site of the *IL4* gene promoter, correlating to IgE production, central to the pathophysiology of asthma [34]. Finally, hypermethylation of the *FOXP3* gene was observed in the regulatory T cells of asthmatic children exposed to high air pollution, as opposed to the ones subjected to low levels [35]. For most of these studies, the assessment of air pollution exposure was performed based either on questionnaires [30], seasonal collecting [31] or on nearby central monitoring sites in the study community [32, 33]. For the latter, in the case of absence of data, the gaps were filled by using data from nearby monitors [32]. Although these studies require further validation in larger samples, they provide an opportunity for reflexion on current practices pertaining environmental assessment.

Such environmental exposure assessments have certain drawbacks. The use of questionnaires, for example, may lead to the misclassification of exposure due to the participant’s difficulty in recalling exposure in a precise way, recall bias or even intentional false reporting of exposure [36]. Seasonal monitoring implies that exposure is only monitored in a discrete time frame, which means that a great deal of data is excluded. Finally, monitoring based on nearby sites also has downsides. Although monitoring can be estimated on a daily basis, gaps in the data monitoring may occur; in addition, this kind of monitoring assumes that the type and degree of exposure is exactly the same for distinct individuals living in the same spatio-temporal setting.

However, sensitive differences in individual exposure might occur that do not coincide with each other and/or with the ones reported for the surrounding environment. For example, a recent study found that the individual exposure to NO_2_ in a group of healthy adults living in Stockholm was significantly different than the one measured for the urban background level (13 versus 20 µg/m^3^). Time-activity patterns such as time spent at home and at the workplace explain part of the variation [37].

In this context, the development of an individual-based assessment of exposure to environmental factors could prove of crucial importance to understanding the dynamics of complex diseases such as asthma [38].

An increasing body of literature has proposed the use of new technologies, like mobile devices in personal health monitoring [39–41] and, in particular, for assessing and self-managing asthma [42, 43]. However, most of these approaches are purely based either on phone calls [42] or text messaging [43] failing to provide a broader context for the disease study. An increasing number of apps for smartphones and tablets aimed to support asthma self-management exist nowadays, but they still lack reliability and accuracy and fail to provide the patient with the comprehensive information about the condition necessary for self-management [44].

Research-wise, mobile devices such as smartphones equipped with a GPS system can provide data on time and location of the patients. In parallel, they can use wireless sensor to record environmental variables (such as the level of humidity or smoke concentration, etc.), and also monitor certain physiological signals to help predict the causes of acute asthma episodes [38].

Examples of both environmental and health personal sensors exist. A research team has recently developed a real-time second and third-hand smoke sensor prototype that measures and records smoke through the adsorption of ambient nicotine vapor [45]. Electrochemical in vivo sensors, which offer near real-time measurement of analytes, have also been improved in the last decades and are now used to detect a range of in vivo targets such as glucose, glutamate, reactive nitrogen species and many neurochemicals [46].

Although there are still some limitations to the use of mobile devices for this particular kind of sensing, such as battery life, wireless coverage [47], and sensor synchronisation [38], technological advances in the area of Ubiquitous Computing are rapidly improving, leading to an endless sphere of applications, with great potential for public health research.

Thus, one of the current goals is the real-time monitoring of the environmental factors that affect disease. From a general perspective, in most gene-environment studies, genotyping has been emphasized in comparison to environmental assessment because genotyping techniques are accurate and systematic, while environmental exposure measures still lack precision [48]. Focusing on this asymmetry, the term “exposome” was coined back in 2005, referring to the concept of assessing one’s life-course environmental exposures (including lifestyle factors) from the prenatal period onwards [49].

Currently, a number of large-scale projects are focused on addressing and developing tools to evaluate the whole environmental exposure and its impact on human health [50–53]. The Human Exposome Project intends to evaluate a number of environmental exposures, from diet to lifestyle and behaviour, combined with genetics and medicine in order to prevent and treat a number of diseases [50]. Similarly, the HELIX project, aims to measure prenatal and postnatal exposure to a variety of chemical and physical variables by using smartphone linked sensors, as well as omics techniques to determine molecular profiles related to exposure [51]. The EXPOsOMICS project, targets to assess the exposome through a personal exposure monitoring system, including sensors, smartphones, geo-referencing and satellites, and will also look into biological samples for internal makers of external exposure, through omics technologies [52]. Finally, the HEALS project focuses on assessing individual exposure to conventional and emerging environmental stressors, using a range of novel technologies, such as mobile phone apps, coupled with DNA sequencing, epigenetic DNA modifications, and gene expression aimed to assess disease phenotypes [53].

The word “-omics” refers to the thorough study of sets of biological molecules with high-throughput techniques providing a more comprehensive analysis of biological systems [54]. Some omics technologies in environmental health include transcriptomics, epigenomics, proteomics and metabolomics [54].

The current focus on the real-time assessment of the exposome, and the inclusion of omics analysis clearly aims for an integrative vision of disease as the result of individual susceptibility to exposure. Rather than the traditional nature versus nurture paradigm, the current model integrates both concepts, leading to a nature *et* nurture coordinated approach, that can significantly advance our understanding of complex non-communicable diseases, like asthma.
